# Unraveling the Origin of the Repulsive Interaction between Hydrogen Adsorbates on Platinum Single-Crystal Electrodes

**DOI:** 10.1021/acs.jpcc.4c05193

**Published:** 2024-08-29

**Authors:** Jinwen Liu, Arthur Hagopian, Ian T. McCrum, Katharina Doblhoff-Dier, Marc T. M. Koper

**Affiliations:** † Leiden Institute of Chemistry, Leiden University, Leiden 2333 CC, The Netherlands; ‡ Department of Chemical and Biomolecular Engineering, Clarkson University, Potsdam, New York 13699, United States

## Abstract

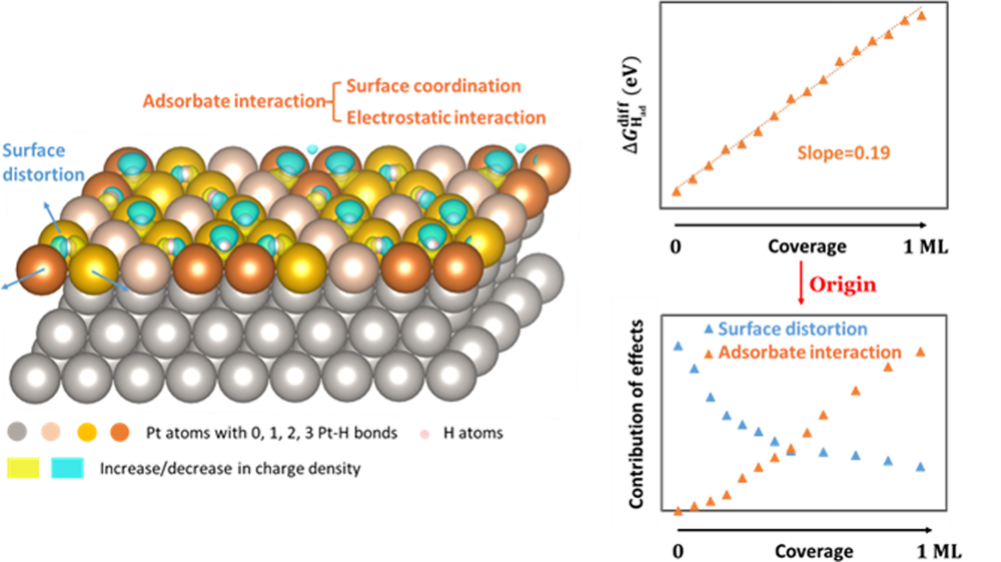

Hydrogen adsorption on platinum (Pt) single-crystal electrodes has been studied intensively in both experiments and computations. Yet, the precise origin and nature of the repulsive interactions observed between hydrogen adsorbates (H_ads_) have remained elusive. Here, we use first-principles density functional theory calculations to investigate in detail the interactions between H_ads_ on Pt(111), Pt(100), and Pt(110) surfaces. The repulsive interaction between H_ads_ on Pt(111) is deconvoluted into three different physical contributions, namely, (i) electrostatic interactions, (ii) surface distortion effect, and (iii) surface coordination effect. The long-range electrostatic interaction, which is generally considered the most important source of repulsive interactions in surface adsorption, was found to contribute less than 30% of the overall repulsive interaction. The remaining >70% arises from the other two contributions, underscoring the critical influence of surface-mediated interactions on the adsorption process. Surface distortion and coordination effects are found to strongly depend on the coverage and adsorption geometry: the effect of surface distortion dominates when adsorbates reside two or more Pt atoms apart; the effect of surface coordination dominates if hydrogen is adsorbed on neighboring adsorption sites. The above effects are considerably less pronounced on Pt(100) and Pt(110), therefore resulting in weaker interactions between H_ads_ on these two surfaces. Overall, the study highlights the relevance of surface-mediated effects on adsorbate–adsorbate interactions, such as the often-overlooked surface distortion. The effect of these interactions on the hotly debated adsorption site for the adsorbed hydrogen intermediate in the hydrogen evolution reaction is also discussed.

## 1 Introduction

Hydrogen (H) adsorption on Pt is one of the most fundamental processes in electrochemistry.^1−6^ On the one hand, H adsorption/desorption on metal surfaces serves as an elementary step for numerous vital electrochemical reactions,^6^ including, but not limited to, hydrogen evolution and oxidation reactions (HER/HOR).^3,6−11^ On the other hand, Pt as an electrode material is not only the best HER catalyst^12^ but also one of the best-studied electrode materials with well-studied single-crystal surfaces.^13^ The use of single-crystal electrodes has facilitated a deeper understanding of electrochemical processes as it allows for straightforward comparisons between theory and experiment.^13−15^ Yet, even for H adsorption on Pt single crystals, captivating questions persist, such as the precise origin and nature of the adsorbate–adsorbate interactions.^3,16,17^

Cyclic voltammetry (CV) provides important information on the kinetics and thermodynamics of various electrochemical processes.^18−20^ The CV of Pt(111) in HClO_4_ solution often serves as a model system since perchlorate anions do not adsorb specifically in the relevant potential window.^21,22^ The CV of Pt(111) in HClO_4_ between ∼0.05 and 0.85 V_RHE_ can be divided into three distinct regions: the H adsorption region (0.05–0.4 V_RHE_), the OH adsorption region (0.55–0.85 V_RHE_), and the double layer region in between (0.4–0.55 V_RHE_).^19^ The broad peak of H_ads_ is typically interpreted using the Frumkin isotherm, which suggests the presence of repulsive interactions between H_ads_ on Pt(111).^23−27^

Since the late twentieth century, numerous research groups have strived to deepen our understanding of H adsorption at the Pt–water interface.^6^ This has involved extensive investigation into the adsorbate–adsorbate interaction for H adsorption on the three basic facets of Pt.^25,28,29^ By fitting a Frumkin isotherm to single-crystal CV data, they find a moderate repulsion between H_ads_ on Pt(111) but weaker interactions on Pt(100) and Pt(110). This repulsive interaction has been confirmed in computational work from Nørskov’s group, which revealed a notable decrease in adsorption strength as the coverage increases from 0 to 1 monolayer (ML) on Pt(111).^30^ The interaction between H_ads_ on Pt(100) was found to be considerably weaker, which is consistent with the experimental results.^25,31^ McCrum and Janik found the same results on Pt(111) and Pt(100), and they also revealed a weak interaction between H_ads_ on Pt(110).^32^

Here, we aim to address two central remaining questions: (i) What is the exact origin of the repulsive interaction for H_ads_ on Pt(111) and (ii) why does the interaction of H_ads_ depend so strongly on the crystal facet of the Pt electrode? We employ a first-principles density functional theory (DFT) approach to probe these questions.

## 2 Methods

### 2.1 Hydrogen Adsorption Energies

The hydrogen adsorption/desorption process can be described by the following reaction
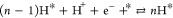
1where *n* is an integer counting the hydrogen atoms adsorbed in the periodic simulation cell, * denotes an adsorption site, H* denotes an adsorbed hydrogen atom, and H^+^ denotes a hydrated proton in solution.

The differential Gibbs free energy of this reaction can be written as^33^
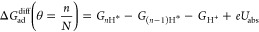
2a
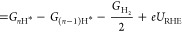
2bwhere the surface coverage θ is defined as the number of adsorbates per number of surface atoms *N* in the supercell. *G*_*n*H*_ is the Gibbs free energy of the surface with *n* hydrogen atoms adsorbed. *G*_H_^+^ and *G*_H_2__ are the Gibbs free energies of H^+^ and H_2_, respectively. *U*_abs_ and *U*_RHE_ refer to the absolute potential and potential with respect to the reversible hydrogen electrode (RHE). The transition from eq 2a to 2b is performed in accordance with the computational hydrogen electrode method.^34^ The effect of electric field on the adsorbate-induced surface dipole is ignored here because previous results^35−38^ and our test calculation (Section S6 in Supporting Information) both indicate that the H atoms adsorb nearly neutrally with a negligible surface dipole.

To compare the free energies in different configurations at the same coverage, we also define the average adsorption energy per adsorbate as^33^
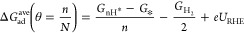
3where G_*_ is the Gibbs free energy of bare surface. The difference between Δ*G*_ad_^diff^ and Δ*G*_ad_^ave^ is discussed in detail in Section S1 of Supporting Information.

Unless otherwise noted, we report all values for Δ*G*_ad_^diff^ and Δ*G*_ad_^ave^ at *U*_RHE_ = 0 V.

To quantify the interaction between H*, the average interaction energies Δ*G*_inter_ are calculated from the change in the average adsorption energy in the case of *n* adsorbates compared to the low coverage limit
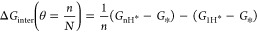
4Note that Δ*G*_inter_ represents the average interaction energy between *n* adsorbed hydrogen atoms, while Δ*G*_ad_^ave^ represents the average Gibbs free energy required to adsorb *n* hydrogen atoms.

The Gibbs free energies for different species are calculated as
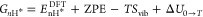
5a
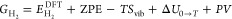
5bwhere  and  are the DFT total energies of the surface with *n* hydrogen atoms adsorbed and the molecular hydrogen, respectively. ZPE is the zero-point energy, *T* = 298 K is the room temperature, *P* is the pressure, *V* is the volume, *S*_vib_ is the vibrational entropy, and  is the temperature correction for internal vibrational energy. The ZPE, *TS*_vib_, and  are calculated in the harmonic approximation, as implemented in the VASPKIT package.^39^ Vibrations are only taken into account for the adsorbate degrees of freedom in the surface normal and parallel directions and in the H_2_ molecule.^5,30,32^ Note that we purposefully do not include configurational entropy in this expression. This makes the expression for *G* compatible with the free energy appearing in the exponent of the Frumkin isotherm (see Section 2.3). Accurately accounting for configurational entropy usually involves a lattice-gas model solved by Monte Carlo simulations.

### 2.2 Computational Details

The calculations were carried out using the Vienna Ab initio Simulation Package (VASP)^40−42^ with a plane-wave basis set. Core electrons were treated using PAW pseudo potentials.^43,44^ Specifically, we used the PAW_PBE pseudopotential dated 04. Feb. 2005 for Pt and the PAW_PBE pseudopotential dated 15. Jun. 2001 for H, which are available from VASP.^43,44^ Unless otherwise specified, the Perdew–Burke–Ernzerhof (PBE)^45^ exchange–correlation functional was employed. This choice of functional is justified for two reasons: First, the change in adsorption energy from 1/16 to 1 ML does not depend sensitively on whether PBE, RPBE, or PBE-vdW-DF is used (see Table S1 and Section S2 in the Supporting Information). Second, from the three functionals tested, PBE captures the adsorption energy  at low coverages best compared to experiment (−0.347 eV from ref (46) and −0.39 eV from ref (47)).

The surfaces were modeled using a four-layer slab with a 4 × 4 unit cell [hexagonal cell for Pt(111), orthogonal cell for Pt(100) and Pt(110)], where the upper two layers were relaxed and the bottom two layers were frozen during optimization to represent the surface and bulk regions, respectively. The lattice constant of bulk Pt was found to be 3.97 Å for the PBE functional, which is in reasonable agreement with the experimental value of 3.92 Å.^48^ Neighboring slabs were separated by 14 Å of vacuum to limit surface–surface interactions.^49^

A 5 × 5 × 1 Monkhorst–Pack mesh,^50^ a plane-wave cutoff energy of 350 eV, and Fermi smearing with a smearing width of 0.05 eV were used. For these settings, differences in the average interaction energy compared to even more converged settings are below 4 meV (i.e., less than 5% of the total value) for θ = 1, as shown in Figure S1.

Note that the present study does not include the effect of solvation. Previous computational results^30,38,51^ as well as our own test calculations using a water bilayer and fully explicit water (Section S3 in Supporting Information) indicate that solvation has a negligible effect on the adsorbate–adsorbate interaction in H adsorption on Pt.

### 2.3 Frumkin Isotherm and Interaction Coefficient

In the mean-field approximation, the H coverage can be described using a Frumkin isotherm^27^
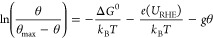
6where θ_max_ is the maximum coverage, *e* is the elementary charge, *k*_B_ is the Boltzmann constant, Δ*G*^0^ is the zero coverage limit of the Gibbs free energy change associated with the global reaction , *U*_RHE_ is the potential on the RHE scale,^27^ and *g* is the interaction coefficient. The left-hand side of this equation describes the change in configurational entropy with changing coverage, and the right-hand side describes the change in Gibbs free energy *G* (excluding configurational entropy) of the system with changing coverage. The interaction coefficient *g* can be obtained from a fit to the CV or computationally from refs (25 and 30)
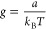
7where *a* is the slope of Δ*G*_ad_^diff^ as a function of coverage θ. A detailed derivation of eq 7 can be found in Supporting Information Section S1.

### 2.4 Average Surface Displacements

The average surface displacements
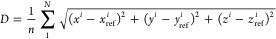
8are used to quantify the surface distortion effect. Here, *x*, *y*, and *z* are the atomic positions in *x*, *y*, and *z* directions. The reference positions (*x*_ref_^*i*^, *y*_ref_^*i*^, and *z*_ref_^*i*^) are set to the positions of the Pt atoms in the optimized bare Pt slab.

### 2.5 Charge Density Analysis

Two kinds of charge density differences are calculated and visualized, namely, the “charge density difference” (Δρ) and the “differential charge density difference” (Δρ_diff_). They differ in the reference system used. The charge density difference Δρ uses the bare surface as the reference, which can be expressed as
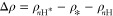
9where  is the charge density of the surface with *n* hydrogen atoms adsorbed, ρ_*_ and ρ_*n*H_ are the charge density of bare surface and of *n* hydrogen atoms, respectively.

The differential charge density difference Δρ_diff_ uses the *n*-1 configuration as reference, which can be expressed as
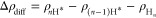
10where ρ_H_*n*__ is the charge density of *n*th H.

## 3 Results and Discussion

### 3.1 H_ads_ on the fcc Site of Pt(111)

At low coverage, on Pt(111), we find that the fcc site is the most favorable site for hydrogen adsorption, although the difference in energy to other sites is low (∼50 meV; see Table 1). The computed adsorption energy at 1/16 ML coverage is −0.333 eV, which is in good agreement with experiment (−0.347 eV)^43^ and DFT calculations (−0.35 eV)^30^ previously reported in the literature. Based on these findings, we exclusively consider the fcc sites on Pt(111) in the subsequent investigations unless otherwise specified.

**Table 1:** Calculations of Δ*G*_ad_ at the Coverage of 1/16 ML H_ads_ on the Different Sites of Pt(111), Pt(100), and Pt(110)

surface	site	Δ*G*_ad_/eV
Pt(111)	top	–0.255
	bridge	–0.285
	**fcc**	**–0.333**
	hcp	–0.283
Pt(100)	Top	–0.282
	**bridge**	**–0.440**
	hollow	–0.182
Pt(110)	top	–0.369
	**short bridge**	**–0.424**
	long bridge	–0.106
	hollow	–0.042

**Figure 1:**
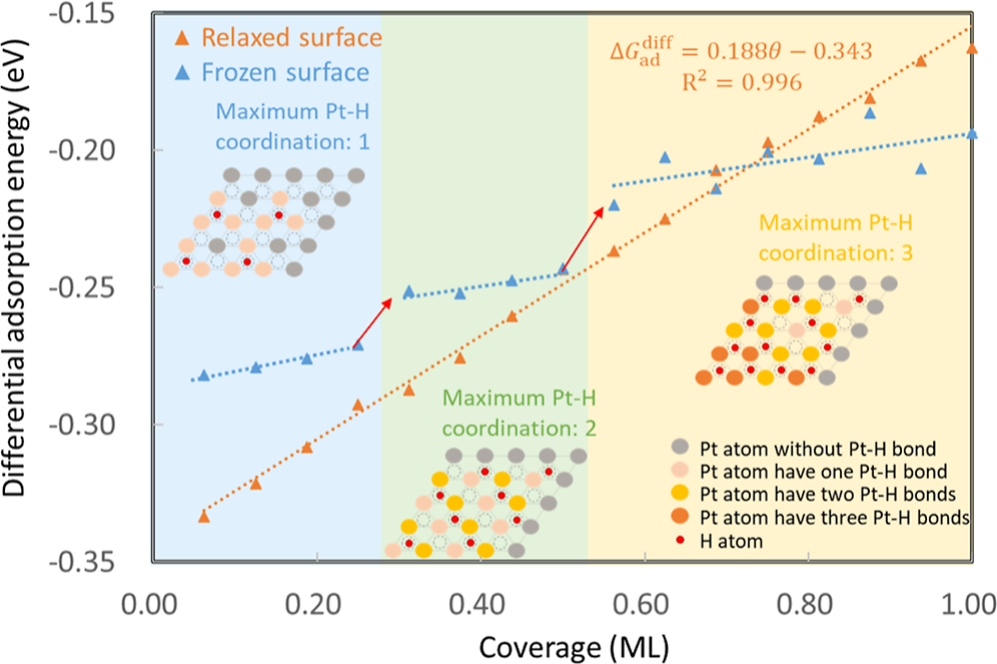
Differential adsorption energy as a function of coverage (ML) for H_ads_ on the fcc site of Pt(111) when keeping adsorbates as far apart as possible (see insets for exemplary configurations). Three regions (blue, green, and yellow) highlight regions with different maximum Pt–H coordination. Red arrows highlight jumps in adsorption energy between these different regions.

To investigate the interaction between H_ads_ at higher coverage, we calculate the coverage-dependent differential adsorption energies (Δ*G*_ad_^diff^). As we expect the H_ads_ to show repulsive interactions,^25,28,30,32^ we initially place the H_ads_ as far apart from each other as possible (see Figure S5d). As shown in Figure 1 (orange triangles), this leads to an adsorption strength (quantified by Δ*G*_ad_^diff^) that decreases nearly linearly with coverage, indicating repulsive interactions between H_ads_, which aligns with previous findings in the literature.^25,30,32^ As Δ*G*_ad_^diff^ appears to depend approximately linearly on the coverage θ, the slope can be directly related to the interaction coefficient *g* (see Supporting Information, Section S1) appearing in the Frumkin isotherm via eq 7. We obtain a slope of 0.188 eV/ML (*g* = 7.3), which is consistent with previous computational results (0.15 eV/ML) by Karlberg et al.^30^ but slightly smaller than the value derived from the experimental results by CV (*g* = 9.6–12.0).^25,28,29^ The discrepancy between our results and those obtained from CV may arise from the error of DFT calculations or exclusion of solvent effects.^52,53^ But even without considering any water molecules, the computed *g* is around 70% of the value obtained from CV. This shows that factors other than the solvation effect dominate the repulsive interactions between H_ads_. In the following, we therefore focus on discussing the origin of these contributions.

To shed more light on the origin of the adsorbate–adsorbate interaction, we further investigate the influence of different configurations on the energetics, including line arrangements (Figure S5a,b), a cluster arrangement (Figure S5c), and a distribution maximizing the distance between H_ads_ (Figure S5d). To compare the energies of different configurations, we make use of the average adsorption energy Δ*G*_ad_^ave^ in Figure S5e. Interestingly, and maybe somewhat unexpectedly, we find the configurations with H atoms arranged in lines to be the most stable ones. However, the average adsorption energy depends only weakly on the configuration, with changes in the average adsorption energy per atom of less than 5 meV for different configurations (see Table S2). This weak dependence of the average adsorption energy on the exact configuration of the adsorbates would be compatible with a long-range interaction between the adsorbates.^26^

A common long-range interaction in surface-adsorbed systems is an electrostatic interaction, often described by the interaction between point-like dipoles. This type of electrostatic interaction is not expected to be strong in the case of H_ads_, however, as H atoms adsorb nearly neutrally with a weak surface dipole only.^35−38^ In fact, in our simulations, we find a dipole moment orthogonal to the surface of less than 0.02 eÅ, leading to a point-dipole–point-dipole interaction that is negligible compared to the changes in binding energy observed in Figure 1 for changing coverage(see estimation in Section S6 in Supporting Information).

**Figure 2:**
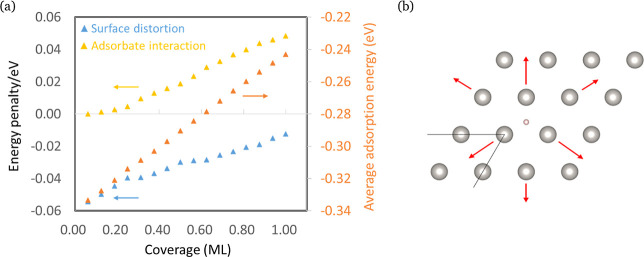
(a) Energy penalties caused by surface distortion, defined as Δ*G*_ad_^ave, relaxed^-Δ*G*_ad_^ave, frozen^, adsorbate interaction, defined as Δ*G*_inter_^frozen^, and average adsorption energy as a function of coverage (ML) for H_ads_ on the fcc site of Pt(111) when keeping adsorbates as far apart as possible. (b) Schematic illustrations of the surface displacement during H adsorption. Gray spheres are the Pt atoms; the rose sphere is the H_ad_. The arrays show the direction of displacement of Pt atoms; their length is proportional to the actual surface movement.

Another candidate for long-range repulsive interactions is the adsorbate-induced surface distortion (relaxation), as discussed in the literature,^24,54−56^ also known as elastic interactions in surface science.^54,57−59^ To study the influence of surface distortion, we perform additional calculations in which all Pt layers are frozen (not allowed to relax throughout DFT cycles), again keeping the adsorbates as far apart as possible. The resulting adsorption strength (as quantified by Δ*G*_ad_^diff^) is shown in the blue data points in Figure 1. Consistent with the literature,^60^ we observe that for the first H_ads_ (1/16 ML), the adsorption strength to the frozen surface decreases compared to the relaxed surface by 52 meV, meaning that the adsorbate on the frozen surface is destabilized compared to the relaxed case. This effect becomes smaller when the coverage increases. The decreasing stabilization of the adsorbates through surface distortion is consistent with the fact that we observe appreciable surface rearrangements during H adsorption at low coverages, which become less substantial as the coverage increases (see Figure S7, orange data). Specifically, the average surface displacement decreases from 0.34 Å at a coverage of 1/16 ML to 0.09 Å at a coverage of 1 ML. This can be rationalized by the fact that at high coverage, the Pt atoms interact with several H_ads_ and cannot rearrange as freely as in the low coverage case. The distortion of surface atoms during H adsorption is visualized in Figure 2b, revealing that the Pt lattice is locally expanded by a single H_ads_. Whenever subsequent H adsorption pushes back on this expansion, its adsorption can be expected to be less favorable. Overall, the results provide evidence that the surface distortion effect contributes significantly to the long-range repulsion between H_ads_. However, the surface distortion cannot be the only effect causing adsorbate–adsorbate interaction for two reasons: First, as shown in Figure 2a, the adsorbate stabilization (negative energy penalty) through surface distortion decreases with increasing coverage, while the adsorption strength shown in Figure 2a (orange data) decreases steadily. Second, if surface distortion were the only effect, Δ*G*_ad_^diff^ would be constant (i.e., a straight, horizontal line) in the case of a frozen surface, which is not the case (see Figure 1, blue data).

In the case of a frozen surface, Δ*G*_ad_^diff^ is characterized by regions with nearly constant adsorption energy, separated by jumps at certain coverages (highlighted by red arrows in Figure 1). These sudden changes in the differential adsorption energy indicate a sudden change in adsorbate–adsorbate interaction at certain coverages and appear to be a consequence of variations in the Pt–H coordination (the number of hydrogen atoms bound to a single Pt surface atom). In the low coverage region (0–0.25 ML), as we place the H_ads_ as far apart as possible, the maximum Pt–H coordination is 1. Above the coverage of 0.25 ML, any newly adsorbed H atom has to interact with at least one Pt atom that is already bonded to another H atom on the surface. When the coverage exceeds 0.5 ML, Pt atoms start bonding with three H_ads_. This coordination effect reduces the adsorption strength of the H atoms at the surface by about 20 meV every time the Pt–H coordination changes.

**Figure 3:**
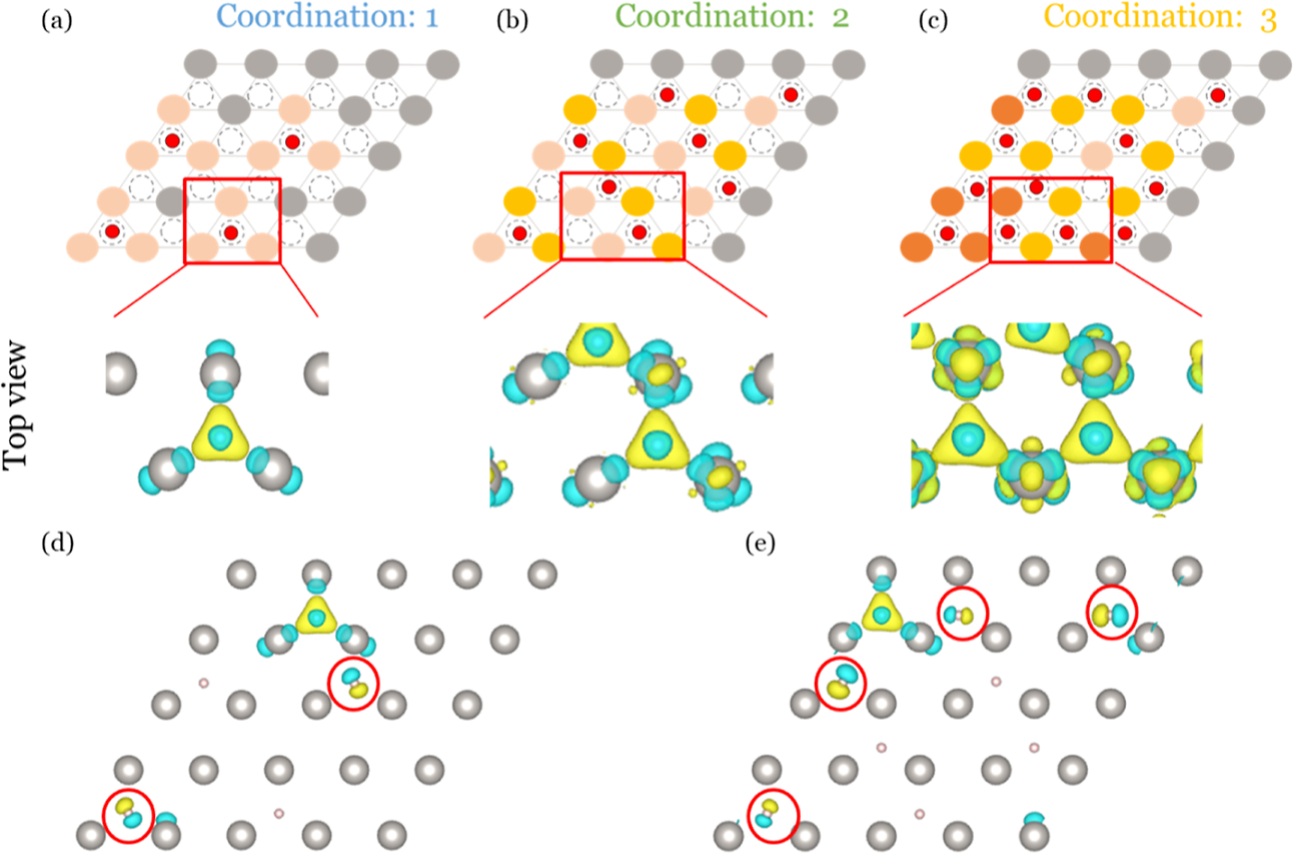
(a–c) Charge density difference plots Δρ at different coverages, as indicated in the figure. (d,e) Differential charge density difference plots Δρ_diff_ when increasing the hydrogen coverage further from θ = 0.25 ML (panel d) and θ = 0.5 ML (panel e). The yellow and blue areas represent increase and decrease in the charge density, respectively. Colors for H and Pt atoms are the same as in Figure 1.

To corroborate the above statement, we visualize the charge density changes (as introduced in Section 2.5) for the three regions in Figure 3a–c. The yellow and blue areas represent increases and decreases in charge density, respectively. When H adsorbs, the charge density moves from the H atom to the adjacent Pt atoms to form the Pt–H bond. Double and triple coordination (see Figure 3b,c) influence this charge transfer, as highlighted by the differential charge density differences plots in Figure 3d,e. As a new H_ad_ adsorbs, it also affects the original H_ads_ close to it, leading to an energy penalty when adsorbing the next H in an adjacent adsorption site on the surface, thereby contributing to the repulsion of H_ads_.

To further support the idea that surface coordination plays a major role once surface distortion effects are excluded, we compare the adsorption energies for different H configurations while keeping the surface frozen. Given the discussion above, we expect that a configuration that requires the platinum atoms to coordinate to several hydrogen atoms, such as when forming a line or cluster, will result in destabilization compared to configurations that place the H_ads_ as far as possible from each other. Figure S6 confirms this: When freezing the surface, the average adsorption strength for line and cluster configurations is indeed smaller than that obtained for H_ads_ placed far apart, corroborating the idea of Pt–H coordination playing a role.

So far, we have shown surface distortion and Pt–H coordination to play an important role in the adsorbate–adsorbate interaction of H_ads_ on Pt(111). However, even if surface distortion is eliminated by freezing the surface and first shell Pt–H coordination effects are excluded by placing hydrogen atoms at least two Pt atoms apart, a small slope in the differential adsorption energy remains (see Figure 1, blue triangles at coverages θ ≤ 0.25 ML), indicating an additional source of interaction. It is tempting to ascribe these remaining interactions to the long-range electrostatic effects between adsorbates already discussed above as these often play a crucial role in surface-adsorbed systems.^54,61,62^ However, the point-dipole–point-dipole interaction expected based on the dipole orthogonal to the surface created by a single H adsorbate is more than 1 order of magnitude too small (<0.1 meV) to explain the change in differential adsorption energy from  ML to  ML for a frozen surface of ∼3 meV (see Figure 1 and the discussion in Supporting Information Section S7). Therefore, either multipole effects or effects of the second coordination shell must be at play in this case. Charge density difference plots shown in Figure S9 in fact show that considerable charge transfer still occurs at Pt atoms not directly coordinated to the H atom, strengthening the assumption that interactions beyond the first Pt–H coordination shell might cause the remaining adsorbate–adsorbate interaction at low coverage. In any case, the remaining interaction is responsible for about 30% of the total repulsive interaction between hydrogen adsorbates on Pt(111), as can be seen when comparing the slope in the differential adsorption energy for a fixed surface at low coverages with the slope obtained for the relaxed surface in Figure 1.

Overall, we have thus identified three different effects causing a change in H adsorption energy with increasing coverage: a contribution from surface distortion, one from first shell coordination effects, and one from long-range effects. In the following, we will denote the latter two contributions as “direct adsorbate–adsorbate interactions”. When increasing the adsorbate coverage while keeping adsorbates as far apart from each other, the surface distortion effect dominates at low coverage, while the direct adsorbate–adsorbate interaction dominates at high coverage (see Figure 2a). But how can it be then that the average adsorption strength in the relaxed case is nearly independent from the adsorbate configuration as shown in Figure S5a? Likely, this is the consequence of surface distortion effects and direct adsorbate–adsorbate interaction balancing each other. This is demonstrated in Figure S6: for line and cluster configurations of the H adsorbates, the adsorbate stabilization through surface relaxation is large (as the surface is relatively flexible in these configurations), while the energy penalty due to direct adsorbate–adsorbate interaction (i.e., on a frozen surface) is also large. For configurations in which the H atoms sit far apart, the opposite is true: adsorbate stabilization through surface distortion and direct adsorbate–adsorbate interaction are both small. Taken together, surface distortion and direct adsorbate–adsorbate interaction thus seem to average out, masking any configurational dependence in the case of a relaxed surface.

In summary, our analysis shows that the repulsion of H_ads_ on Pt(111) is not the result of a single effect. Instead, there are three different effects that contribute roughly equally: (i) a long-range surface distortion effect, (ii) a short-range surface coordination effect, and (iii) a long-range multipole or surface coordination effect. The strongly linear coverage-dependent differential adsorption energy in the case of a relaxed surface is caused by the (fortuitous) balance between these effects.

### 3.2 H_ads_ on the Top Site of Pt(111)

**Figure 4:**
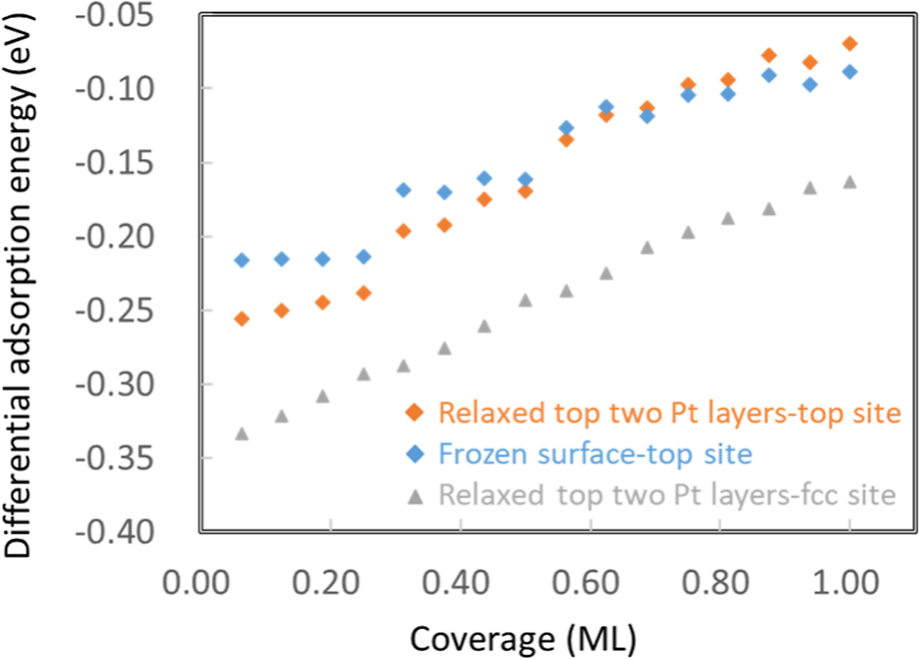
Comparison of differential adsorption energy as a function of coverage (ML) between H adsorption on the top site in the case of relaxed two top Pt layers (orange rhombus) and a frozen surface (blue rhombus) and on the fcc site in the case of relaxed two top Pt layers (gray triangle).

The comparison between H adsorption on top and fcc sites of Pt(111) is interesting because both spectroscopic results^8^ and DFT calculations^10^ revealed that there is a transition from H adsorption on the fcc site to the top site as the potential shifts negatively (or the coverage increases), and the latter has been considered as the intermediate for HER.^11,63^ Here, we investigate the energetic difference between H_ads_ on the top site and fcc sites within the coverage region between 0 and 1 ML. Figure 4 shows that H adsorption at the fcc site (gray triangle) is more energetically favorable than that at the top site (orange rhombus) by approximately 0.1 eV in the entire coverage region.

Interestingly, the relationship between differential adsorption energy and coverage for H adsorption on the top site delineates three distinct regions, even in the relaxed case, in contrast to its fcc counterpart. Moreover, the results for the top site in the relaxed case (orange rhombus) are similar to those in the frozen case (blue rhombus). On the basis of these observations, we conclude that the effect of surface coordination dominates in the whole coverage region between 0 and 1 ML for H_ads_ on the top site, as corroborated by the results that the surface distortion effect is much weaker for H_ads_ on the top site in the low coverage region (see Figure S10). This lack of surface distortion is because hydrogen atoms adsorbed in on-top sites sit above only one Pt atom, resulting in less horizontal displacement. The charge density analysis in Figure S11 is also in agreement with the single surface coordination in the coverage region between 0 and 1 ML. However, the effect of second coordination shell becomes prevalent when the coverage exceeds 0.25 ML, and intensifies further beyond 0.5 ML, as illustrated in Figure S12. These phenomena account for the energy jumps near these coverages for on-top H adsorption, as depicted in Figure 4.

Based on the fact that H_ads_ on the fcc site is always more favorable than on top sites (independently of coverage), we further estimate the probability for H adsorption occurring on the top sites using Boltzmann distribution. From the coverage of 10/16 ML, the population ratio for a state with one H_ads_ residing in an on-top site and the rest in fcc sites compared to a configuration in which all H_ads_ reside in fcc sites is about 0.7% (see Figure S13). Considering that there should be several such configurations, we expect an on-top coverage of a few percent, consistent with what has been reported before.^64^ These on-top H_ads_ may be the intermediates for the HER,^8,10^ and the low coverage of the surface with on-top hydrogens could explain why the measured Tafel slopes suggest the adsorbate species to be a minority species on the surface.^31^

### 3.3 H_ads_ on Pt(100) and Pt(110)

Equipped with comprehensive insight into H_ads_ interactions on Pt(111), we examined the coverage-dependent H adsorption also for the other two low-index facets of Pt, Pt(100), and Pt(110). In the context of this work, and to allow a simple comparison to the results on Pt(111), we do not consider surface reconstructions here^65^ nor the simultaneous adsorption of OH.^32,66^ The results for these facets should therefore not be quantitatively compared to experiments, and we focus on qualitative trends only.

As presented in Table 1, the most stable sites for Pt(100) and Pt(110) are identified as the bridge and short bridge sites, respectively, which are more energetically favorable than other sites by about ∼200 meV. This site preference is more pronounced than for Pt(111) and is consistent with previous results reported in the literature.^30,67−69^ The differences in site preference between Pt(111), Pt(100), and Pt(110) are determined by both geometric and electronic structures of these surfaces.^70^ The balance between Pt–H coordination and Pt–H distance likely leads to the optimal overlap between the H 1s orbital and Pt 5d orbitals at the fcc site of Pt(111) but at bridge sites of Pt(100) and Pt(110).

**Figure 5:**
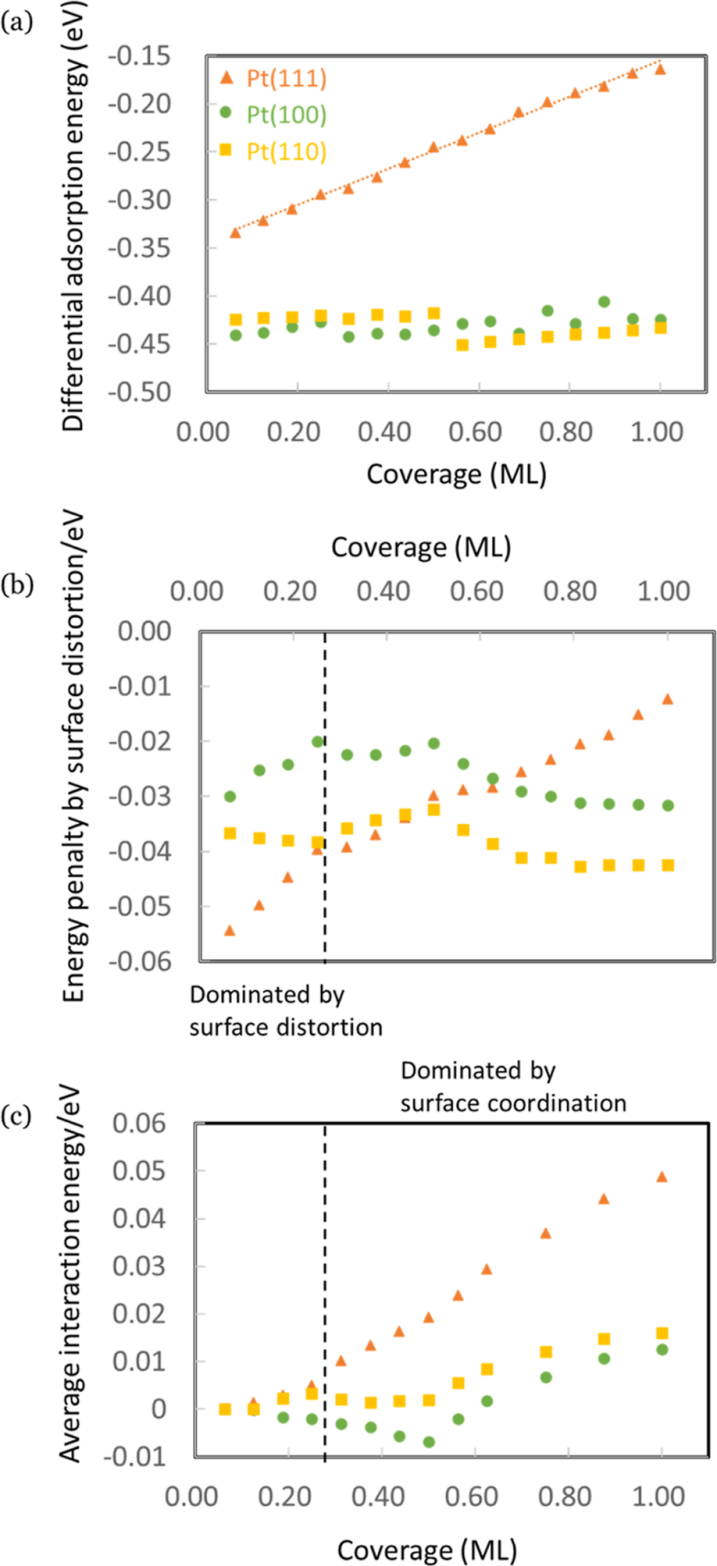
Comparison of (a) differential adsorption energy, (b) energy penalties caused by surface distortion, and (c) average interaction energy as a function of coverage (ML) between H adsorption on Pt(111) (orange triangles), Pt(100) (green circles), and Pt(110) (golden squares). Figures (a,b) were calculated with the top two Pt layers relaxed. Figure (c) was calculated for the frozen surface.

To investigate the surface-dependent adsorbate interaction between H_ads_, we again compared the coverage-dependent differential adsorption energies for these three facets of Pt. Similarly to what we did on Pt(111), we thereby increase the coverage such that adsorbates are placed as far apart as possible. Consistent with the results in the literature,^27,29,30,32^ we show in Figure 5a that the differential adsorption energy for Pt(100) and Pt(110) exhibits negligible coverage dependence, which implies minimal interactions between H_ads_ on these two surfaces. We rationalize the results by comparing the surface distortion effect and surface coordination effect for these three facets by means of their energy penalties at different coverages (defined in Section 3.1), as shown in Figure 5b,c, respectively. In the low coverage region, the surface distortion effect is dominant but is less significant on Pt(100) and Pt(110) than on Pt(111) (see Figure 5b). We expect this difference to be caused by Pt(111) being a more compact surface and H_ads_ adsorbing in the 3-fold (fcc) site on Pt(111) while adsorbing in 2-fold (bridge) sites on Pt(100) and Pt(110). In the high-coverage region, the surface coordination effect becomes dominant, again similar but much less significant on Pt (100) and Pt(110) compared to that on Pt(111) (Figure 5c). The charge density analysis for these three surfaces also supports that the surface coordination effect is much more intense on Pt(111) than on the other two surfaces at the same coverage, as shown in Figure S14. We attribute this again to the more open structure (less H_ads_ per surface area) and different site preference (less surface coordination per adsorbate) of Pt(100) and Pt(110) compared to Pt(111). In conclusion, the substantially reduced interaction between H_ads_ on Pt(100) and Pt(110) arises from their more open surface and different site preference compared to Pt(111), which results in a weaker surface distortion effect in the low-coverage region and a significantly weaker surface coordination effect in the high-coverage region.

### 3.4 Discussion on Model Implications and Limitations

The detailed insights into repulsive interactions and preferred adsorption sites of H adsorption on Pt(111), Pt(100), and Pt(110) surfaces revealed by our first-principles DFT study shed light on the understanding of surface adsorption processes and mechanisms for the electrocatalytic reaction involving H adsorption. Specifically, our findings delineate the complex interplay of electrostatic interactions, surface distortion effects, and surface coordination effects in dictating H adsorption behavior. Although our study is mainly focused on the H adsorption behavior of underpotential deposition (UPD) region of Pt,^7^ these insights also set a foundation for the further understanding of the transition between H_upd_ and overpotential deposition of hydrogen (H_opd_) and the HER mechanism. Besides, the origin of the coverage-dependent adsorption energy for H adsorption could extend to other adsorbates that serve as reactive intermediates in various electrocatalytic reactions. By management of surface distortion and surface coordination effects, it may be possible to drive improvements in catalyst activity and selectivity. On the other hand, limitations and possible extensions of our model should also be addressed. First, the surface reconstruction^65^ and the presence of other adsorbates,^20,32^ which may significantly alter the adsorption behavior, especially on Pt(100) and Pt(110), are not considered here. Second, although we showed that water molecules may play a secondary role in the overall repulsion between H_ads_, the presence of water may have a non-negligible influence on the preferred adsorption site,^52^ the adsorption entropy,^25^ and the (coverage-dependent) surface distortion. Studying these effects requires thermodynamic sampling of water, which will be interesting to investigate in the future.

## 4 Conclusions

In this study, we used a first-principles DFT approach to deconvolute the origin of the repulsive interaction for H_ads_ on platinum single-crystal electrodes into different physical contributions, specifically, (i) electrostatic interaction, (ii) surface distortion effect, and (iii) surface coordination effect. The electrostatic interaction was found to contribute a maximum of 30% of the overall repulsive interaction and not to be caused by simple dipole–dipole interactions. The remaining >70% of the adsorbate–adsorbate repulsion arises from surface distortion and Pt–H coordination effects, underscoring the critical influence of surface-mediated interactions on the adsorption process. Furthermore, these effects are coverage- and adsorption geometry dependent and balancing with each other: the effect of surface distortion dominates when adsorbates reside two or more Pt atoms apart; the effect of surface coordination dominates if hydrogen is adsorbed on neighboring adsorption sites, which result in the nearly linear relation between the adsorption energy and coverage. Similar conclusions can be drawn for Pt(100) and Pt(110), though they are considerably less pronounced compared to Pt(111). Finally, although we find the fcc site to be the most favorable site on Pt(111), independent of coverage, we estimate that a few percent of H_ads_ might be present on the top site at coverages obtained around 0 V vs RHE. Top site hydrogen may thus well be the minority species expected to play a role as the catalytic intermediate in the HER.

Although we have verified that the presence of water does not dominate the H–H interaction, it will be interesting in future work to address the additional influence of water at the surface on the adsorption strength at different sites, the surface distortion, and the adsorption entropy.

## Supporting Information Available

Difference between differential and average adsorption energy and their relation with the interaction coefficient; test calculations for the choice of DFT functional; effect of water on H adsorption; configuration effect for H_ad_ on Pt(111); surface displacements of Pt(111), Pt(100), and Pt(110) during H adsorption; electrostatic interaction between H_ad_; surface distortion effect for H_ad_ on the top site of Pt(111); charge density difference analysis for H_ad_ on the top site of Pt(111); probability for H adsorption occurring on the top sites; and comparison of charge density difference analysis for Pt(111), Pt(100), and Pt(110) (PDF)
