# Supplementary material for: Unraveling the Origin of the Repulsive Interaction between Hydrogen Adsorbates on Platinum Single-Crystal Electrodes
Source: J Phys Chem C Nanomater Interfaces. 2024 Aug 29;128(36):15019–28. doi: 10.1021/acs.jpcc.4c05193 (PMC11403660; doi:10.1021/acs.jpcc.4c05193)
Supplement: Supplementary file 1 — jp4c05193_si_001.pdf [file jp4c05193_si_001.pdf]

Supporting information to

Unraveling the Origin of the Repulsive Interaction between Hydrogen Adsorbates on Platinum Single-Crystal Electrodes

Jinwen Liu[1], Arthur Hagopian[1], Ian T. McCrum[2], Katharina Doblhoff-Dier[1*], Marc T. M. Koper[1*]

[1] Leiden Institute of Chemistry, Leiden University, 2333 CC, Leiden, The Netherlands

[2] Department of Chemical and Biomolecular Engineering, Clarkson University, Potsdam New York 13699, United States of America

* Corresponding authors: m.koper@chem.leidenuniv.nl; k.doblhoff-dier@lic.leidenuniv.nl

1. Difference between $\Delta G_{\mathbf{ad}}^{\mathbf{diff}}$ and $\Delta G_{\mathbf{ad}}^{\mathbf{ave}}$ and their relation with g

The differential and average adsorption energy ($\Delta G_{\mathrm{ad}}^{\mathtt{diff}}$ and $\Delta G_{\mathrm{ad}}^{\mathrm{ave}}$) given in Eqs. 2-3 in the main text and repeated here, are defined here as in the text by Aravind and Janik,[1]

$$\Delta G_{\mathrm{ad}}^{\mathtt{diff}} = G_{n\mathrm{H}^*} - G_{(n-1)\mathrm{H}^*} - \frac{G_{\mathrm{H}_2}}{2} + eU_{\mathrm{RHE}}, \tag{S1a}$$

$$\Delta G_{\mathrm{ad}}^{\mathrm{ave}} = \frac{G_{\mathrm{nH}^*} - G_*}{n} - \frac{G_{\mathrm{H}_2}}{2} + eU_{\mathrm{RHE}}, \tag{S1b}$$

where all variables are defined in the main text.

In the Frumkin isotherm, the (potential-dependent) Gibbs free energy of adsorption is assumed to increase linearly with coverage according to[2]

$$\Delta G_{\mathrm{ad}} = \Delta G_{\mathrm{non-interact}} + k_b T \cdot g \cdot \theta,$$

where g is the interaction parameter, k_B the Boltzmann constant and T the temperature. In this notation, g is positive for repulsive interaction. Finally, ΔG_{ad} and $\Delta G_{\mathrm{non-interact}}$ are the (standard) free energy of adsorption for interacting and non-interacting (Langmuir-type) adsorbates. From the above expression, it becomes clear that g is related to the (constant) slope a of the adsorption Gibbs free energy ΔG_{ad} via

$$g = \frac{1}{k_B T} a, \tag{S2}$$

where a is the slope of ΔG_{ad} as a function of coverage.

Considering that we defined two different adsorption energies ($\Delta G_{\mathrm{ad}}^{\mathtt{diff}}$ and $\Delta G_{\mathrm{ad}}^{\mathrm{ave}}$) in equations (S1a) and (S1b), it may, however, not be immediately clear which of these quantities should enter equation (S2) in order to determine the interaction parameter g computationally. In the following, we derive the Frumkin isotherm to clarify the (approximate) connection between $\Delta G_{\mathrm{ad}}^{\mathtt{diff}}$ and g. This connection will become exact in the limit of a large number of possible adsorption sites N_{site} and a

large number of adsorbates n considered.

We start by considering a system with n hydrogen adsorbates and (m-n) protons in solution. The total Gibbs free energy (including the configuration entropy) of this system is given by

$$G^{\mathrm{tot}}(n) = G_{n\mathrm{H}^*} + (m-n)G_{\mathrm{H}^+} - (m-n)eU_{\mathrm{abs}} - TS_{\mathrm{conf}}(n), \quad \text{(S3a)}$$

$$= G_{n\mathrm{H}^*} + (m-n)\frac{G_{\mathrm{H}_2}}{2} - (m-n)eU_{\mathrm{RHE}} - TS_{\mathrm{conf}}(n). \quad \text{(S3b)}$$

The transition from eq. S3a to S3b is performed in accordance with the computational hydrogen electrode (CHE) method.[3] For large enough number of adsorption sites N_{site}, the configuration entropy can be approximated as[4]

$$S_{\mathrm{conf}}(n) = -N_{\mathrm{site}}k_{\mathrm{B}}[\theta \ln \theta + (1-\theta)\ln(1-\theta)], \quad \text{(S4)}$$

where N_{site} is the number of available adsorption sites and $\theta = n/N_{\mathrm{site}}$ is the coverage.

When plotting $G^{\mathrm{tot}}(n)$ as a function of potential U_{RHE}, in a finite supercell, one obtains plot as shown below.

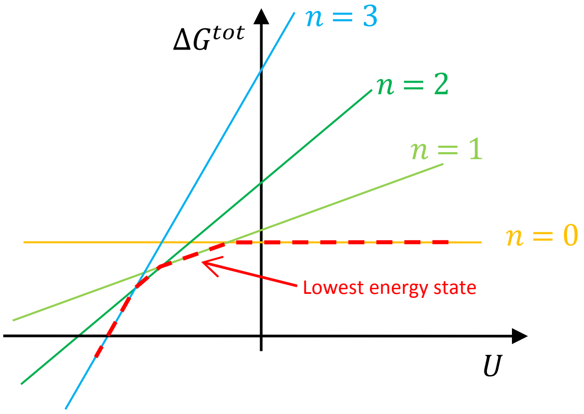

Note: This plot assumes repulsive interaction between adsorbates.

The transition from a state with $n-1$ adsorbates to the state with n adsorbates, will occur at the potential $U_{\mathrm{RHE}}^{(n-1)\to n}$ at which the Gibbs free energy of the two states are in equilibrium, leading to

$$G_{n\mathrm{H}^*} - G_{(n-1)\mathrm{H}^*} - \frac{G_{\mathrm{H}_2}}{2} + eU_{\mathrm{RHE}}^{(n-1)\to n} = TS_{\mathrm{conf}}(n) - TS_{\mathrm{conf}}(n-1). \tag{S5}$$

In a finite system, the coverage will change promptly from $n-1$ to n as the potential decreases from above $U_{\mathrm{RHE}}^{(n-1)\to n}$ to a value below. If n and N_{site} are large (for ever-increasing supercell sizes), the coverage $\theta = n/N_{site}$ can be changed smoothly, by changing U_{RHE}.

We can now try to simplify Eq. (S5). First, we recognize the left hand side of Eq. (S5) as the expression for $\Delta G_{\mathrm{ad}}^{\mathrm{diff}}$. In the limit of large numbers, the right hand side can be further approximated as

$$\frac{S_{\mathrm{conf}}(n) - S_{\mathrm{conf}}(n-1)}{1} \approx \frac{\mathrm{d}S_{\mathrm{conf}}}{dn} = \frac{\mathrm{d}S_{\mathrm{conf}}}{d\theta}\frac{d\theta}{dn} = -k_{\mathrm{B}}\ln\left(\frac{\theta}{1-\theta}\right). \tag{S6}$$

Combing eq (S5)-(S6), we arrive at

$$\ln\left(\frac{\theta}{1-\theta}\right) = -\frac{G_{n\mathrm{H}^*} - G_{(n-1)\mathrm{H}^*} - \frac{G_{\mathrm{H}_2}}{2} + eU_{\mathrm{RHE}}}{k_{\mathrm{B}}T} = -\frac{1}{k_{\mathrm{B}}T}\Delta G_{\mathrm{ad}}^{\mathrm{diff}}. \tag{S7}$$

Comparing this with the Frumkin isotherm within the Nernstian approximation,[5,6]

$$\ln\left(\frac{\theta}{1-\theta}\right) = -\left(g\theta + b + \frac{eU_{\mathrm{RHE}}}{k_{\mathrm{B}}T}\right), \tag{S8}$$

Where b is a constant that depends on the equilibrium potential at zero coverage, we find

$$\Delta G_{\mathrm{ad}}^{\mathrm{diff}} = (k_B T \cdot b + eU_{\mathrm{RHE}}) + k_B T g \cdot \theta . \tag{S9}$$

Hence, if the system under investigation can be described via a Frumkin isotherm, $\Delta G_{\mathrm{ad}}^{\mathrm{diff}}$ should be (at least approximately) a linear function of the coverage θ, and g can be extracted from a linear fit $\Delta G_{\mathrm{ad}}^{\mathrm{diff}} = C + a\theta$ between $\Delta G_{\mathrm{ad}}^{\mathrm{diff}}$ and θ (at $U_{\mathrm{RHE}} = 0$ V or any other potential) as

$$g = \frac{a}{k_{\mathrm{B}}T}, \tag{S10}$$

which is equivalent to Eq. 7 in the main text and a is the slope of $\Delta G_{\mathrm{ad}}^{\mathrm{diff}}$ as a

function of θ.

For H adsorption on Pt(111), we indeed find a nearly perfect linear slope (see Figure. 1 in the main text), justifying the extraction of an interaction parameter g from the slope of $\Delta G_{\mathrm{ad}}^{\mathrm{diff}}$.

2. Test calculations for the choice of DFT functional

The average interaction energies at the coverage of 1 ML, $\Delta G_{\text{inter}}(\theta = 1)$, were evaluated to check the influence of the choice of density functional on the results. As $\Delta G_{\text{inter}}(\theta = 1)$ captures the change in adsorption energy with coverage, its stability (with respect to different functionals used and different parameters used in the DFT setup) is expected to be a good indicator for the convergence of the adsorbate – adsorbate interaction (as a function of coverage) that we are interested in in this paper.

As shown in Table S1, the adsorption energy obtained with PBE in the low coverage limit is in better agreement with experimental data. Values obtained for ΔG_{inter} $(\theta = 1)$ are similar for all three functionals, suggesting that results obtained for the adsorbate-adsorbate interaction (i.e., the change in adsorption energy with coverage) should be robust towards the choice of functional.

Figure S1 shows convergence tests for $\Delta G_{\text{inter}}(\theta = 1)$ as a function of the number of k-points used to sample the Brillouin zone, the plane-wave energy cutoff energy and the smearing width σ. The values used to compute final results are highlighted in red. As can be seen in Figure S1, further converging the k-point grid, the plane-wave energy cutoff or the smearing width, will not change $\Delta G_{\text{inter}}(\theta = 1)$ by more than 5% of its absolute value, suggesting that the setting used are sufficiently converged to allow for a meaningful interpretation.

Table S1. Calculated adsorption energy at the coverage of 1/16 ML, $\Delta G_{\mathrm{ad}}(\theta = 1/16)$, and the average interaction energies at the coverage of 1 ML, $\Delta G_{\mathrm{inter}}(\theta = 1)$, as obtained with three different DFT functionals. The computational setup which has been used for these test calculations correspond to: 4×4×4 Pt slab in an orthogonal cell, 5×7×1 k-points, plane wave cutoff energy of 450 eV. The remaining values are equivalent to those given in the methods section in the main text.

Functionals	$\Delta G_{\mathrm{ad}}(\theta = 1/16)$ (eV)	$\Delta G_{\mathrm{inter}}(\theta = 1)$ (eV)
PBE	-0.335	0.092
RPBE	-0.184	0.088
PBE-vdW-DF	-0.261	0.091
	ΔG_{ad}	
Experiment (Ref. 7)	-0.39	
Experiment (Ref. 8)	-0.347	

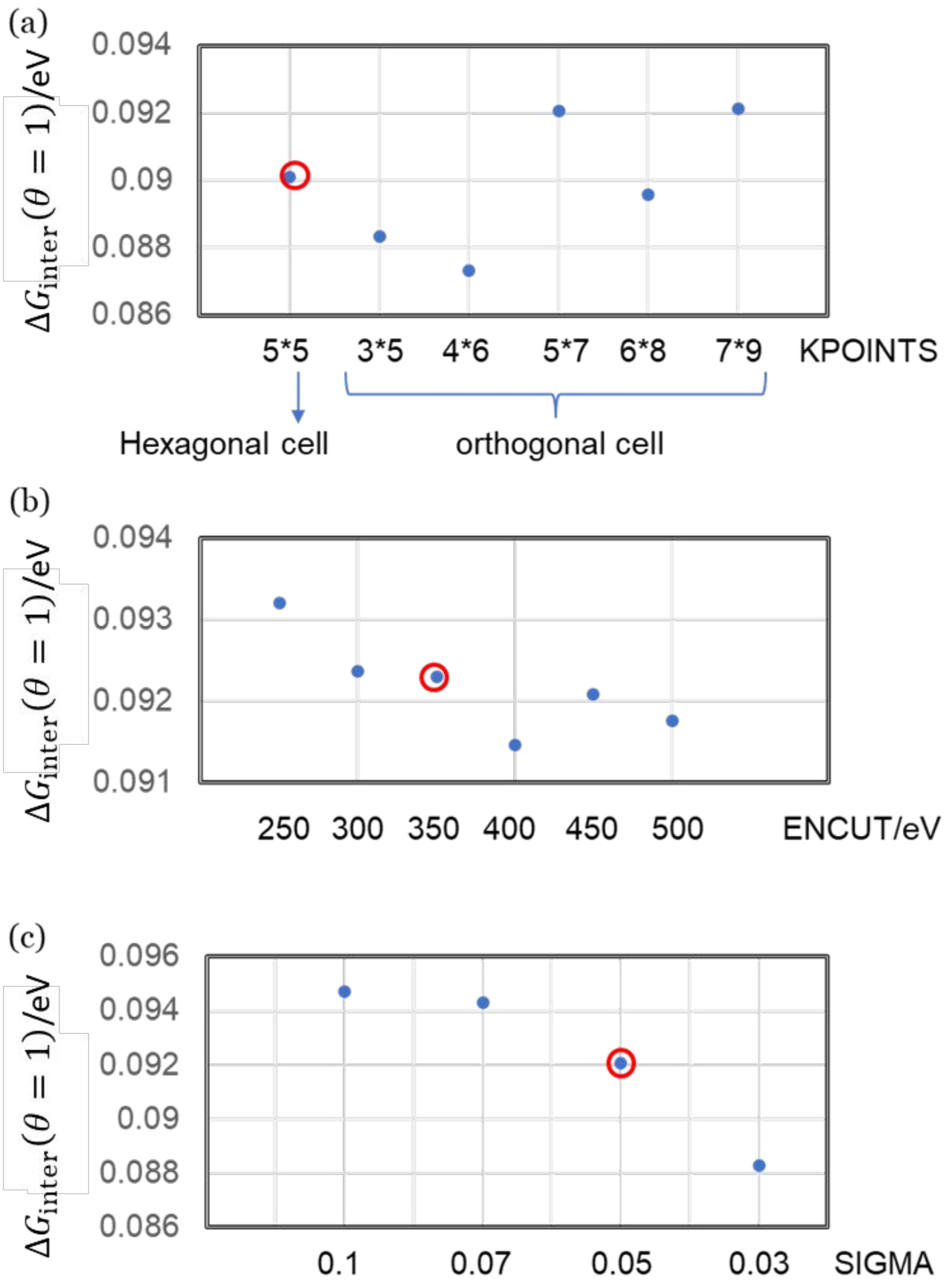

Figure S1. Parameters convergence analysis in DFT calculations. (a) k-point grid (where $a * b$) denotes a k-point grid of $a \times b \times 1$) (b) plane wave cut-off energy, (c) smearing width. Unless otherwise mentioned, we use a 4×4×4 orthogonal Pt cell, a 5×7×1 k-point grid, a plane wave cutoff energy of 450 eV, and a smearing width of 0.05 eV.

3. Effect of water on H adsorption

Water bilayer

To investigate the effect of water on H adsorption, we first consider a four-layer slab with a 3×3 hexagonal Pt unit cell and a water bilayer, as shown in Figure S2a-d. The 3×3 supercell was chosen as it is commensurate with a hexagonal water bilayer as considered here. The differential adsorption energy of H adsorption with water bilayer is calculated by

$$\Delta G_{\mathrm{ad}}^{\mathrm{diff}} = G_{n\mathrm{H}^*+\mathrm{H_2O}} - G_{(n-1)\mathrm{H}^*+\mathrm{H_2O}} - \frac{G_{\mathrm{H_2}}}{2} + eU_{\mathrm{RHE}}, \tag{S10}$$

where $G_{n\mathrm{H}^*+\mathrm{H_2O}}$ is the Gibbs free energy of the surface with the water bilayer and n hydrogen atoms adsorbed. Other quantities are defined in the main text.

As shown in Figure S3a, the water bilayer has negligible effect on the overall slope of $\Delta G_{\mathrm{ad}}^{\mathrm{diff}}$ as a function of coverage. The water bilayer does, however, influence $\Delta G_{\mathrm{ad}}^{\mathrm{diff}}$ at intermediate coverages, with small energy jumps at 2/9, 5/9 ML and 6/9 ML. We associate these jumps with an energy cost to push the water molecules away from the surface as we see the average Pt – water distance increasing with coverage (see Figure S2c-d). Clearly, these jumps would influence some of the conclusions made in our paper. However, these jumps may easily be overly pronounced in a (static) water bilayer description of the water; in more realistic representations of the adjacent water, this effect should be attenuated. As the average behavior follows the trends observed without a water bilayer, we conclude that the water bilayer has negligible effect on the overall interaction between H_{ad}. The slight increase in differential adsorption energy in the region between 0 ML and 0.6 ML, may, however, explain the slight deviations between the interaction parameter g extracted from cyclic voltammograms in the region above 0 VRHE (corresponding to 0 ML to ~2/3 ML coverage) and the g values extracted from the computational results.

Fully explicit water

We also investigate the water effect on H adsorption in a 4×5×4 orthogonal Pt unit cell with a fully explicit water model using ab initio molecular dynamics (AIMD), as shown in Figure S2e-f. The AIMD calculations were performed using CP2K/ Quickstep package using PBE-D3 as exchange-correlation function, the DZV-MOLOPT-SR-GTH basis set, a plane wave cutoff of 300 eV, a time step of 1 fs, and a Nosé–Hoover thermostat. The average adsorption energy of H adsorption with fully explicit water is calculated by

$$\Delta G_{\mathrm{ad}}^{\mathrm{ave}} = \frac{\langle G_{\mathrm{Pt}+n\mathrm{H}^*+\mathrm{H_2O}}\rangle_t - \langle G_{\mathrm{Pt}+\mathrm{H_2O}}\rangle_t}{n} - \frac{G_{\mathrm{H_2}}}{2} + eU_{\mathrm{RHE}}, \quad \text{(S11)}$$

where $\langle G_{\mathrm{Pt}+n\mathrm{H}^*+\mathrm{H_2O}}\rangle_t$ is the time averaged Gibbs free energy of the surface with fully explicit water and n hydrogen atoms adsorbed from our molecular dynamics runs. Other quantities are defined in the main text. The average adsorption energies are calculated and compared here because the AIMD calculations were only performed for several representative coverages (0, 0.2, 0.4, 0.6, 1 ML).

As shown in Figure S3b, the fully explicit water model does not have an obvious effect on the overall slope of $\Delta G_{\mathrm{ad}}^{\mathrm{ave}}$ as a function of coverage – at least not within the error bars achievable within a 15ps molecular dynamics run, when extracting snapshots every 50 fs for analysis.

Since the fully explicit water model still does not show an obvious effect on the overall trend of coverage dependent adsorption energy, and since the interaction parameter g that we find without including water differs only by 30% from the experimentally determined value,[2] we ignore the effect of water in the following.

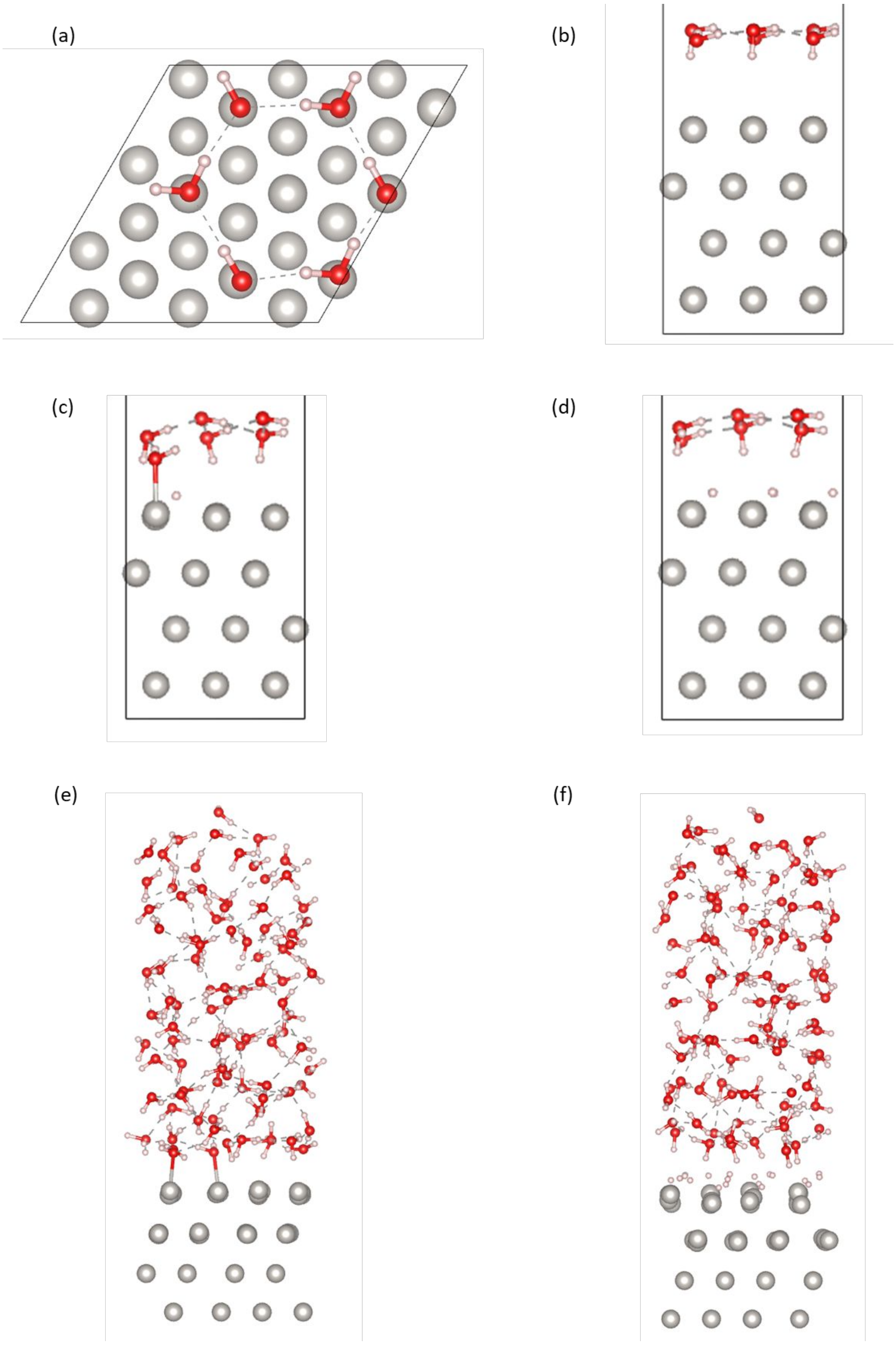

Figure S2. (a) top and (b) side view of 3×3×4 hexagonal Pt unit cell with 1 water bilayer. Equilibrium snapshots for 3×3×4 hexagonal Pt unit cell with 1 water bilayer and (c) 1/9 ML, (d) 5/9 ML H_{ad}. Equilibrium snapshots for 4×5×4 orthogonal Pt unit cell with fully explicit water molecules and (e) 0 ML and (f) 1 ML H_{ad}.

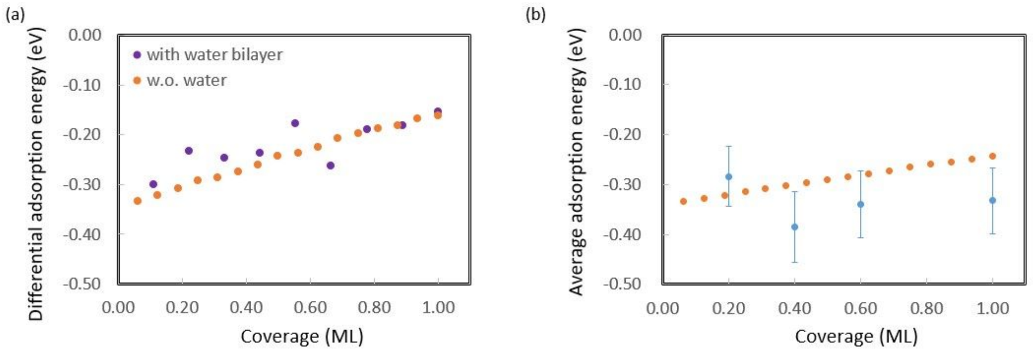

Figure S3. (a) Comparison of the differential adsorption energy as a function of coverage for the system without water and with water bilayer (b) Comparison of the average adsorption energy as a function of coverage for the system without water and with fully explicit water.

4. Configuration effect for H_{ad} on Pt(111)

To investigate the effect of the exact configuration of the adsorbed hydrogens on the surface, several different hydrogen configurations have been tested. In Fig. S4, we show the different (symmetry inequivalent) configurations for 2 hydrogens adsorbed on a 4x4 surface unit cell. In Fig. S5, we schematically show different configurations considered at higher coverages, namely placing H adsorbates in rows (Figures. S5a and S5b), in a pulk (Figure. S5c) and as far apart as possible (Figure. S5d). In the “row” configurations, once one row is filled, the next H atoms are placed in a second row, that can either be adjacent to the first (Figure. S5a) or as far from the first line as possible (Figure. S5b).

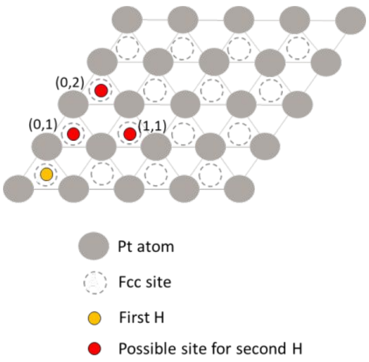

Figure S4. Schematic of 3 different configurations for two H adsorbates on Pt(111) surface.

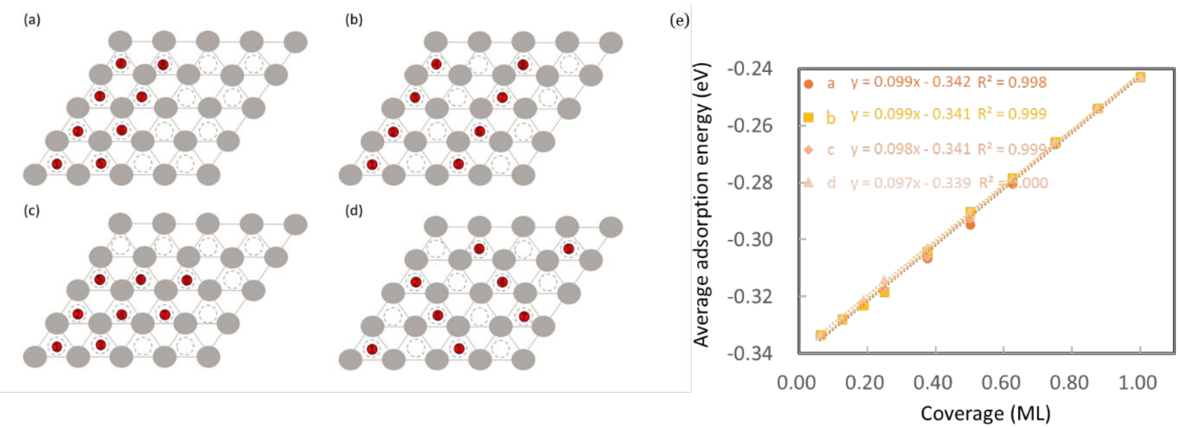

Figure S5. (a)-(d) Schematic of 4 different configurations for 8 H adsorbates on Pt(111) surface. (e) Average adsorption energy as a function of coverage (ML) for configurations a-d when relaxing the surface. Dotted lines are linear regressions of the plotted data.

Relaxed surface

Figure. S5e shows the resulting average adsorption energies $\Delta G_{\mathrm{ad}}^{\mathrm{ave}}$when relaxing the Pt surface as a function of coverage for the configurations considered. Table S2 summarized the numerical values.

Table S2: Configurations effect on $\Delta \mathrm{G}_{\mathrm{ad}}^{\mathrm{ave}}$ in different coverage of H_{ad} on Pt(111) surface.

Coverage	Sites/configurations	$\Delta \mathrm{G}_{\mathrm{ad}}^{\mathrm{ave}}/\mathrm{eV}$
2/16 ML	**(0,1)**	**-0.328**
	(0,2)	-0.327
	(1,1)	-0.325
4/16 ML	**One line**	**-0.318**
	One diagonal line	-0.313
	**Close**	**-0.318**
	Far apart	-0.314
6/16 ML	**Two close lines (with 4 and 2 atoms)**	**-0.307**
	Two far lines (with 4 and 2 atoms)	-0.304
	Close	-0.306
	Far apart	-0.303
8/16 ML	**Two close lines**	**-0.295**
	Two far lines	-0.290
	Close	-0.293
	Far apart	-0.290
10/16 ML	**Three close lines (with 4, 4 and 2 atoms)**	**-0.280**
	Three close lines (with 4, 2 and 4 atoms – considered config. b in Fig. S5e)	-0.278
	Close	-0.279
	Far apart	-0.278
12/16 ML	**Three close lines**	**-0.267**

	Close	-0.266
	Far apart	-0.266
14/16 ML	Close	-0.254
	Far apart	-0.254
1 ML	-	-0.243

Frozen surface

A similar study is performed while keeping the Pt atoms frozen. As shown in Figure. S6, the exact configuration has a much larger influence on the average adsorption energy $\Delta G_{\mathrm{ad}}^{\mathrm{ave}}$ in this case.

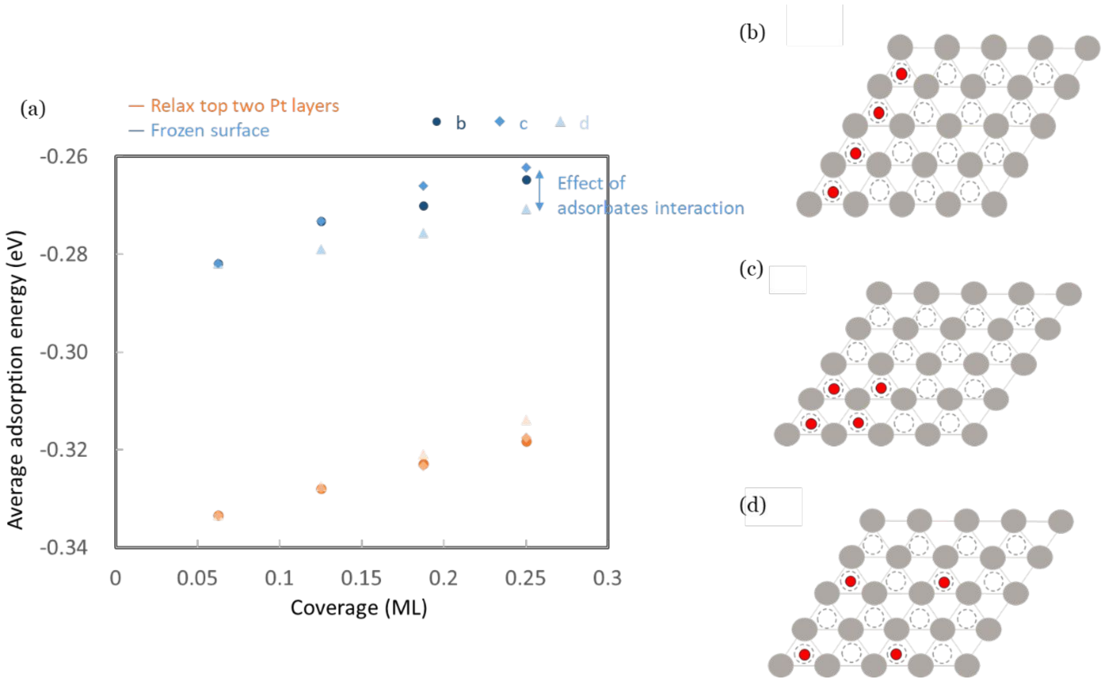

Figure S6. (a) Average adsorption energy as a function of coverage in configuration (b) forming line, (c) cluster, and (d) placing the H_{ads} as far as possible from each .

5. Surface displacements of the Pt(111), Pt(100), Pt(110) during H adsorption

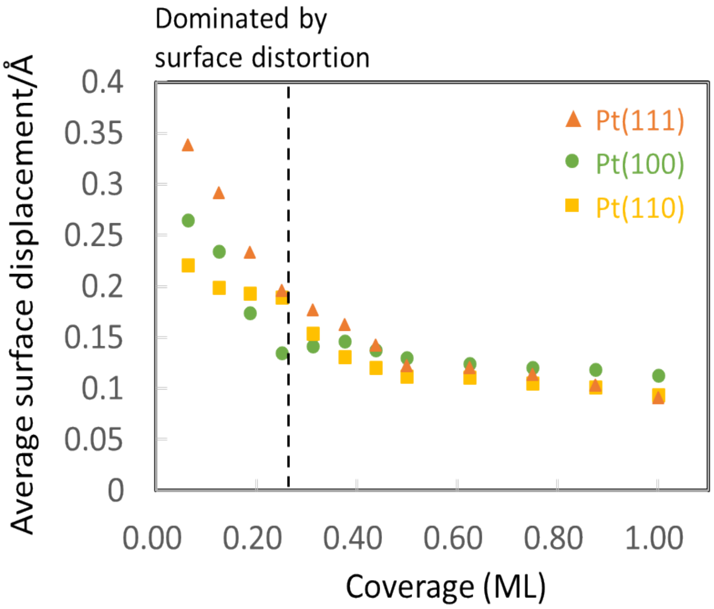

Figure S7. Comparison of surface displacements as a function of coverage (ML) between H adsorption on Pt(111) (orange triangles) Pt(100) (green circles) and Pt(110) (golden squares).

The lattice displacement upon adsorption of a single hydrogen atom occurs mainly in x,y direction (i.e, parallel to the surface), as sketched in Figure. S7. The long arrows correspond to a displacement of $\Delta r = 0.05$ Å, the short arrows to $\Delta r = 0.03$ Å. Smaller displacements are not shown.

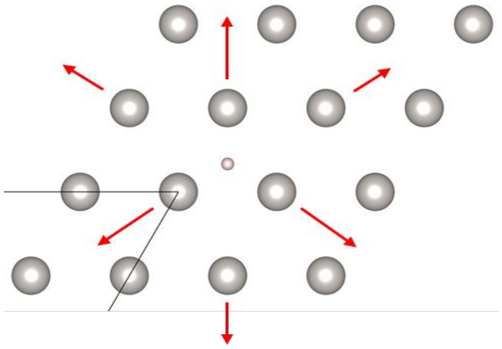

Figure S8. Lattice displacement of the Pt(111) surface during H adsorption. Grey spheres are the Pt atoms, red sphere is the H_{ad}. The arrays show the direction of displacement of Pt atoms, the arrow length indicates how far the atoms move (see text above).

6. Electrostatic interaction between H_{ad}

The adsorption of hydrogen atoms at the Pt surface causes charge transfer, as shown in Figure. S8 for the case of two adsorbed hydrogen atoms. This charge transfer can lead to a dipole moment. For a single H atom at the Pt(111) surface, we find a dipole moment of $\mu_z =$ 0.013 eÅ from the dipole correction applied in the DFT calculations. (Note that the dipole correction was not used throughout the paper, but only for this particular test).

In the following, we show that this dipole moment cannot be responsible for the adsorbate - adsorbate interaction observed at low coverages for a frozen surface. To this end, we approximate the adsorbate – adsorbate interaction by only a dipole – dipole interaction. In the far-field approximation, the interaction of two (parallel) dipoles in vacuum is given by

$$\Delta E_{\mathrm{dipole-dipole}} = \frac{\mu_1 \mu_2}{4\pi\varepsilon_0 r^3}, \tag{S11}$$

where μ_1 and μ_2 are the two dipole moments, ε_0 the permeability of vacuum, and r the distance between two dipoles. In the following we consider the interaction energy of one dipole placed at $(x,y) = \left(\frac{L_x}{2,0}\right)$ (where L_x is the x-dimension of the supercell) with all periodically repeated images of a second dipole at $(x,y) = (0,0)$.

Setting $\mu_1 = \mu_2 = \mu_z =$ 0.013 eÅ and considering the size of the supercell, this gives

$$\Delta E^{\mathrm{tot}}_{\mathrm{dipole-dipole}} = 0.04 \text{ meV},$$

considerably smaller than the remaining adsorbate – adsorbate interaction that can be found from Figure. 1 in the main text.

Several comments are due at this point: First, as shown in Figure. S8, the charge density difference is quite extended in space. A point dipole and the far field approximation will thus be a poor approximation. Second, the charge density difference will not only give rise to a dipole moment, but also to multipole moments,

which could be considerably larger, leading to a larger interaction. The point of the exercise above is solely to prove that a dipole – dipole interaction, as often considered for adsorbate – adsorbate interactions, is not sufficient to explain the long-range adsorbate – adsorbate interaction between H adsorbates observed on frozen Pt surfaces.

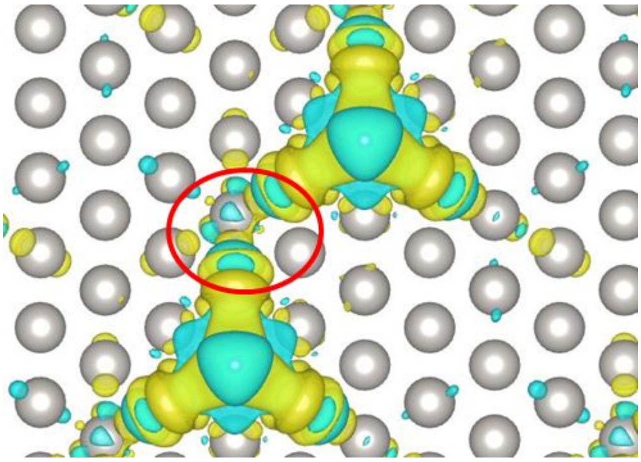

Figure S9. Top view of charge density differences ($\Delta\rho = \rho_{2\mathrm{H}^*} - \rho_{*} - \rho_{2\mathrm{H}}$) for the surface with 2 H_{ad} ($\theta = 2/16$ ML) put far apart with each other. The grey and small red sphere are Pt and H atom, respectively. The yellow and blue sphere represent increasing and decreasing charge density, respectively. Red circle indicates the region in which the charge density difference caused by the adsorption of two different H atoms overlaps.

7. Surface distortion effect for H_{ad} on top site of Pt(111)

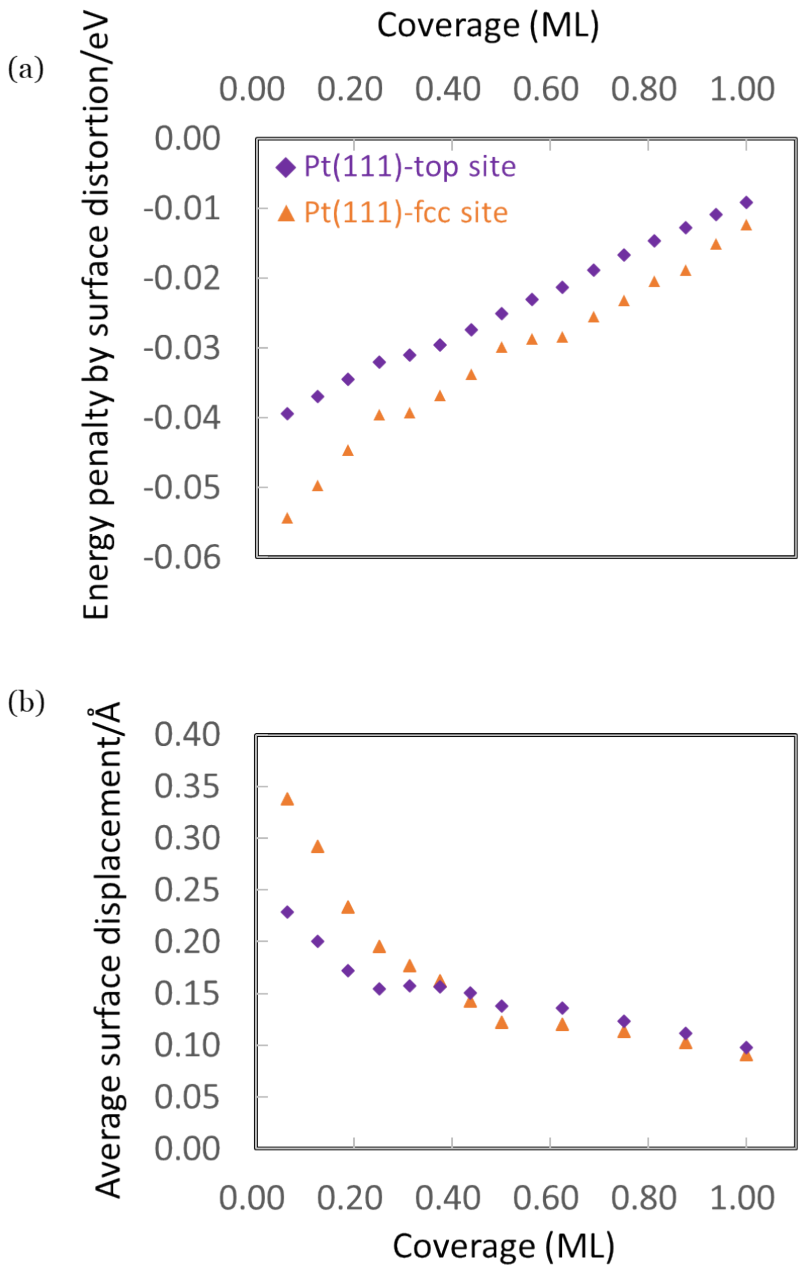

Figure S10. Comparison of (a) energy penalty caused by surface distortion and (b) average surface displacement as a function of coverage (ML) between H adsorption on top site (puple rhombus) and fcc site (orange triangle).

8. Charge density difference analysis for H_{ad} on top site of Pt(111)

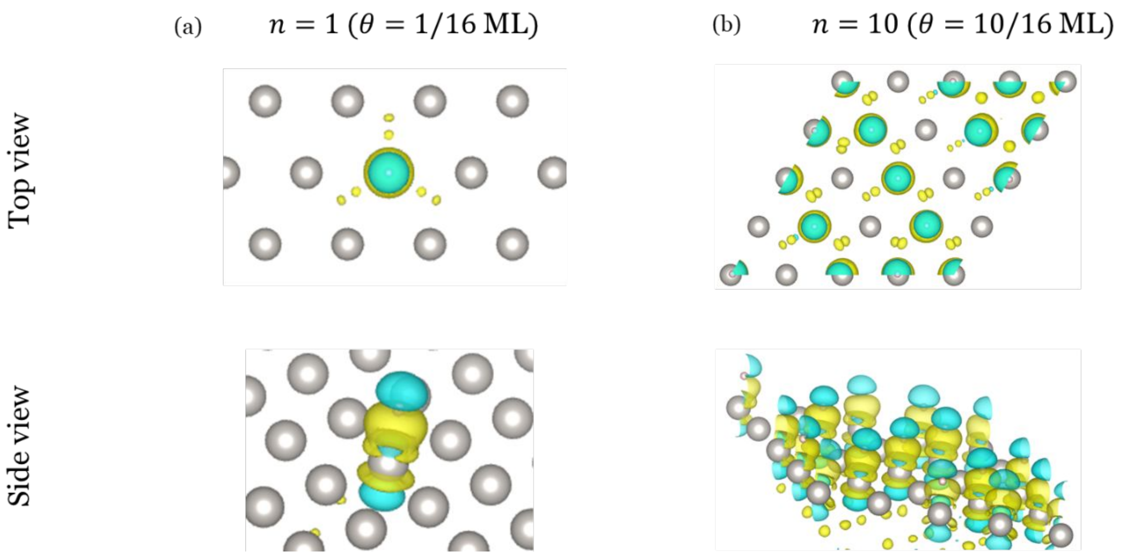

Figure S11. Top and side views of charge density differences between Pt surface with n H_{ad} and bare surface, n= (a) 1 and (b) 10. Yellow iso-surfaces denote an increase in charge density; blue iso-surfaces a decrease in charge density.

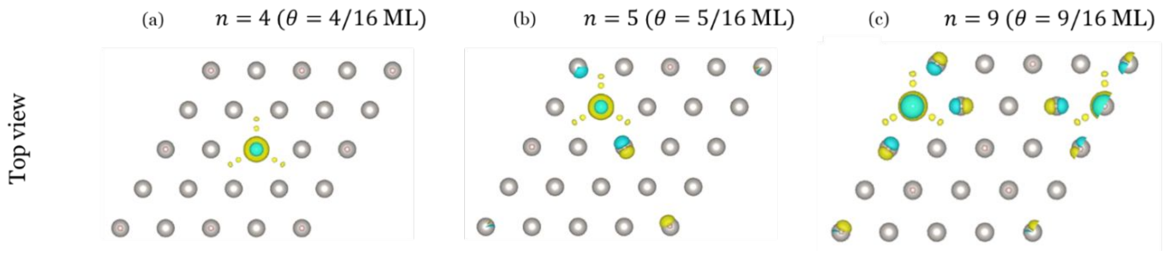

Figure S12. Top view of charge density differences between Pt surface with n H_{ad} and Pt surface with $(n-1)$ H_{ad}, $n =$ (a) 4, (b) 5, (c) 9. Yellow iso-surfaces denote an increase in charge density; blue iso-surfaces a decrease in charge density.

9. Probability for H adsorption occurring on the top sites

We approximate the ratio of populating a configuration in which one hydrogen atom is adsorbed in a top site versus populating a state in which all hydrogen atoms are in fcc sites, as

$$P(1\ \mathrm{top}) = \frac{e^{-\frac{G_2}{k_b T}}}{e^{-\frac{G_1}{k_b T}}} = e^{-\frac{\Delta}{k_b T}}, \tag{1}$$

where $G_1$and G_2 are the Gibbs free energy for x H_{ad} on fcc sites and (x-1) H_{ad} on fcc sites and 1 H_{ad} on top site, respectively. $\Delta = G_2 - G_1$.

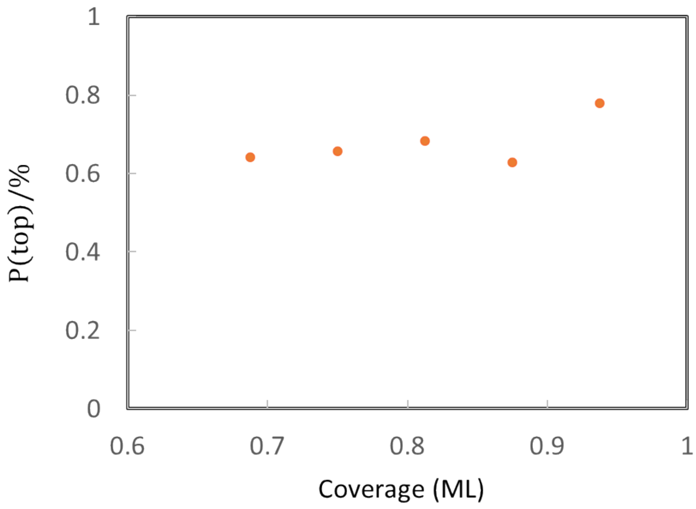

Figure S13. Probability for H adsorption occurring on the top sites.

10. Comparison of charge density difference analysis for Pt(111), Pt(100) and Pt(110)

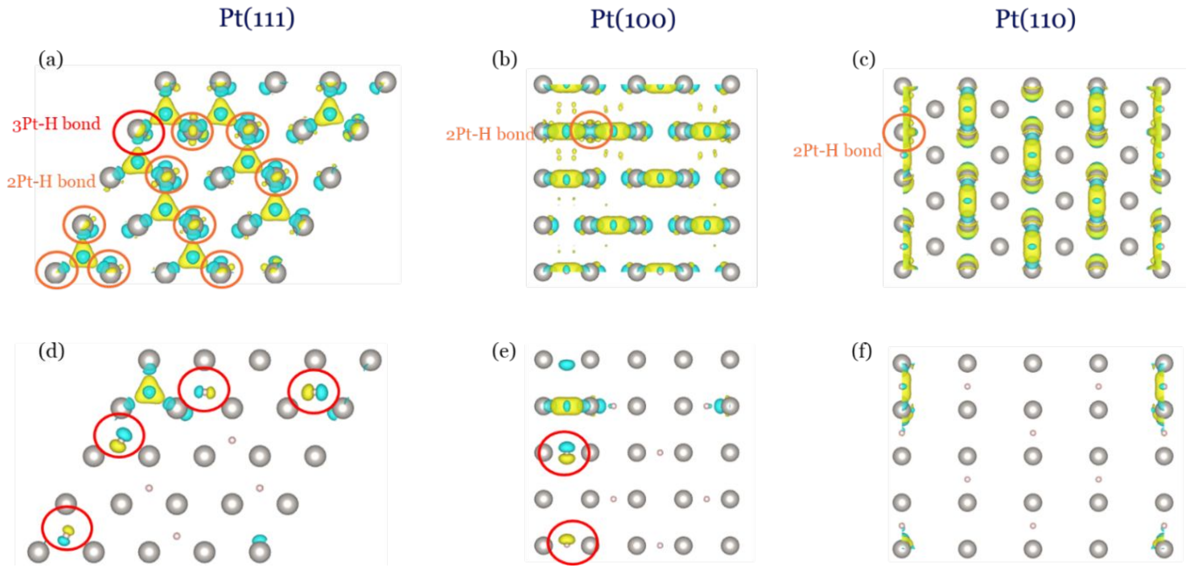

Figure S14. Top view of charge density differences between Pt surface with 9 H_{ad} ($\theta = 9/16$ ML) and bare surface for (a) Pt(111), (b) Pt(100) and (c) Pt(110). Top view of charge density differences between Pt surface with 9 H_{ad} ($\theta = 9/16$ ML) and with 8 H_{ad} ($\theta = 8/16$ ML) for (c) Pt(111), (d) Pt(100) and (e) Pt(110). Yellow iso-surfaces denote an increase in charge density; blue iso-surfaces a decrease in charge density.

References

(1) Asthagiri, A., Janik, M. J., *Computational Catalysis*; Eds.; RSC catalysis series; RSC Publishing: Cambridge, UK, 2014.

(2) Bard, A. J.; Faulkner, L. R. *Electrochemical Methods: Fundamentals and Applications*, 2nd ed.; Wiley: New York, 2001.

(3) Nørskov, J. K.; Rossmeisl, J.; Logadottir, A.; Lindqvist, L.; Kitchin, J. R.; Bligaard, T.; Jónsson, H. Origin of the Overpotential for Oxygen Reduction at a Fuel-Cell Cathode. *J. Phys. Chem. B* **2004**, *108* (46), 17886–17892. https://doi.org/10.1021/jp047349j.

(4) Skúlason, E.; Karlberg, G. S.; Rossmeisl, J.; Bligaard, T.; Greeley, J.; Jónsson, H.; Nørskov, J. K. Density Functional Theory Calculations for the Hydrogen Evolution Reaction in an Electrochemical Double Layer on the Pt(111) Electrode. *Phys. Chem. Chem. Phys.* **2007**, *9* (25), 3241–3250. https://doi.org/10.1039/B700099E.

(5) Gómez, R.; Orts, J. M.; Álvarez-Ruiz, B.; Feliu, J. M. Effect of Temperature on Hydrogen Adsorption on Pt(111), Pt(110), and Pt(100) Electrodes in 0.1 M $HClO_4$. *J. Phys. Chem. B* **2004**, *108* (1), 228–238. https://doi.org/10.1021/jp034982g.

(6) Garcia-Araez, N.; Climent, V.; Feliu, J. M. Analysis of Temperature Effects on Hydrogen and OH Adsorption on Pt(111), Pt(100) and Pt(110) by Means of Gibbs Thermodynamics. *Journal of Electroanalytical Chemistry* **2010**, *649* (1–2), 69–82. https://doi.org/10.1016/j.jelechem.2010.01.024.

(7) Koeleman, B. J. J.; De Zwart, S. T.; Boers, A. L.; Poelsema, B.; Verhey, L. K. Adsorption Study of Hydrogen on a Stepped Pt (997) Surface Using Low Energy Recoil Scattering. *Nuclear Instruments and Methods in Physics Research* **1983**, *218* (1–3), 225–229. https://doi.org/10.1016/0167-5087(83)90983-3.

(8) Norton, P. R.; Davies, J. A.; Jackman, T. E. Absolute Coverage and Isostemc Heat of Adsorption of Deuterium on Pt(111) Studied by Nuclear Microanalysis. *Surface Science* **1982**, *121* (1), 103–110. https://doi.org/10.1016/0039-6028(82)90239-4.
